# AIM 2 inflammasomes regulate neuronal morphology and influence anxiety and memory in mice

**DOI:** 10.1038/srep32405

**Published:** 2016-08-26

**Authors:** Pei-Jung Wu, Hsin-Yu Liu, Tzyy-Nan Huang, Yi-Ping Hsueh

**Affiliations:** 1 Graduate Institute of Life Sciences, National Defense Medical Center, Taipei 114, Taiwan; 2 Institute of Molecular Biology, Academia Sinica, Taipei 115, Taiwan

## Abstract

Inflammasomes are the protein assemblies that consist of inflammasome sensors, adaptor apoptosis-associated speck-like proteins containing a CARD (ASC) and inflammasome caspase. Inflammasomes sense multiple danger signals via various inflammasome sensors and consequently use caspase to trigger proteolytic processing and secretion of IL-1β cytokines. Recent studies have suggested that neurons use their own innate immune system to detect danger signals and regulate neuronal morphology. Here, we investigate whether inflammasomes, the critical components of innate immunity, participate in regulation of neuronal morphology and function. Among various sensors, *Absent in melanoma 2* (*Aim2*) expression in neurons is most prominent. Adding synthetic double-stranded DNA (dsDNA) to cultured neurons induces IL-1β secretion in an AIM2-dependent manner and consequently downregulates dendritic growth but enhances axon extension. The results of *Aim2* knockout and knockdown show that AIM2 acts cell-autonomously to regulate neuronal morphology. Behavioral analyses further reveal that *Aim2*−/− mice exhibit lower locomotor activity, increased anxious behaviors and reduced auditory fear memory. In conclusion, our study suggests that AIM2 inflammasomes regulate neuronal morphology and influence mouse behaviors.

Innate immunity is a naive defense system that neutralizes and removes various foreign pathogens. It also acts as an alarm system to respond to intrinsic danger signals, such as stress and neurodegeneration^1^,^2^. In addition, immune responses are highly associated with neurodevelopmental disorders, such as autism spectrum disorders and schizophrenia^3^,^4^,^5^,^6^. In brains, microglial cells are recognized as the specialized immune cells that respond to infection and neurodegeneration^7^,^8^,^9^. However, neurons have also been shown to express critical molecules that are involved in innate immune responses, such as Toll-like receptors (TLRs) and the downstream adaptors myeloid differentiation primary response protein 88 (MYD88), the Toll/IL-1 receptor (TIR) domain-containing adaptor inducing IFN-β (TRIF) and the Sterile α and TIR motif–containing protein 1 (SARM1), as well as inflammasomes^10^,^11^,^12^,^13^,^14^. Neurons also produce inflammatory and antiviral cytokines, as well as corresponding receptors for these cytokines^13^,^15^. Overall, neurons have their own innate immunity, which responds to endogenous ligands^1^,^16^ to regulate neurogenesis, neural differentiation and neurodegeneration^13^,^16^,^17^,^18^,^19^.

Among various TLRs, TLR7 has been extensively studied in the nervous system. TLR7 recognizes single-stranded RNAs, including bacterial and viral RNAs, as well as endogenous mRNAs and miRNAs^1^,^16^,^20^,^21^. TLR7 activation delivers a signal to MYD88 and c-FOS, thereby inducing IL-6 expression to restrict axon and dendrite outgrowth of cultured neurons through both autocrine and paracrine signaling mechanisms^13^,^16^. Interestingly, TLR7 is activated in neuronal cultures in the absence of exogenous ligand. RNAs released from dead cells or miRNAs released through exosomes likely play roles in TLR7 activation of cultured neurons^13^,^16^,^19^. Echoing the role of TLR7 in neural differentiation, exploratory activity of *Tlr7* knockout mice is altered at the juvenile stage^13^.

In addition to IL-6, neuronal TLR7 activation also increases the level of *Il-1*β mRNA expression, but the level of secreted IL-1β proteins in the supernatants of cultured neurons is not increased^13^. Cytoplasmic pro-IL-1β must be cleaved by an inflammasome—a composite assembly that consists of inflammatory caspases, adaptor apoptosis-associated speck-like proteins containing a CARD (ASC) and various sensors to detect inflammation-inducing stimuli^22^ —to produce and secrete the mature form of IL-1β^23^,^24^,^25^,^26^,^27^. Different inflammatory sensors detect various secondary signals, rather than the signals that activate TLRs, to activate inflammasomes^27^,^28^. It is likely that a proper secondary signal is necessary to activate neuronal inflammasomes in order to induce IL-1β processing in neurons. The inflammasome sensors belong to two families: the nucleotide-binding oligomerization domain-like receptor (NLR) family and the PYHIN (hematopoietic interferon-inducible nuclear antigens with 200-amino-acid repeat-domain-containing protein) family. Among these various sensors, NLRP3 is the most studied inflammasome sensor in brain^29^,^30^,^31^,^32^,^33^. NLRP1, NLRC4 and AIM2 are also involved in brain injury and the innate immune response in brain^14^,^31^,^34^,^35^. Other sensors, such as NLRP6 and NLRP12, have been less extensively studied^36^,^37^. AIM2 is a member of the PYHIN family, while NLRP1, NLRP3, NLRP6, NLRP12 and NLRC4 all belong to the NOD-like receptor family^22^. In this report, we explore whether neurons are able to sense stimulation and activate inflammasomes and investigate whether, similar to TLRs, inflammasome activation regulates neuronal morphogenesis and mouse behaviors.

## Results

### Aim2 is expressed in developing neurons

**Figure 1:**
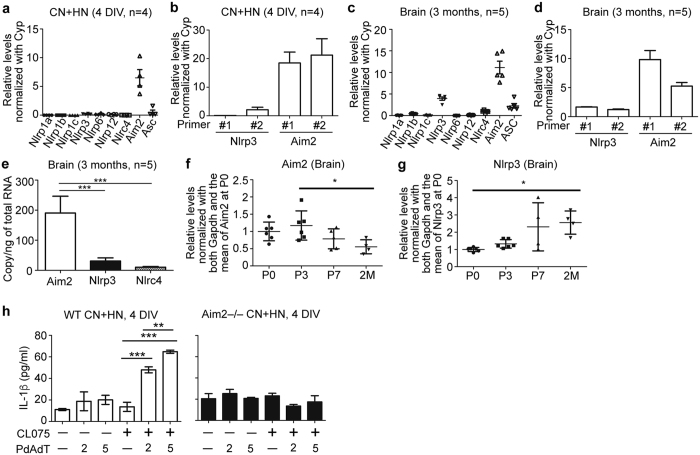
*Aim2* is expressed in neurons and is involved in IL-1β secretion. (**a**–**g**) Expression of a series of inflammasome-related genes in mouse cortical and hippocampal mixed cultures (CN + HN) at 4 days *in vitro* (DIV) and in mouse brains, examined by quantitative-PCR using both the Universal ProbeLibrary (**a**,**c**,**e**,**f,g**) and the SYBR Green (**b**,**d**) systems. The ages of examined mice are indicated. In cultured neurons, *Cyp* was used as internal control. In brains, *Gapdh* was used as internal control because its expression levels were more constant in brains of different ages. (**e**) Absolute copy number of *Aim2*, *Nlrp3* and *Nlrc4* transcripts in adult brains. (**h**) Activation of TLR7 and AIM2 induces IL-1β secretion. Cortical and hippocampal mixed cultures were pretreated with CL075 (6 μM) to activate TLR7 for 6 h and then transfected with poly dAdT (0, 2 or 5 μg) for a further 4 h. The levels of secreted IL-1β in cultured media were measured using ELISA. (**h**) left, WT; right, *Aim2*−/−. The data are presented as the mean ± s.d. *p < 0.05; **p < 0.01; ***p < 0.001. The experiments were repeated independently three times.

To investigate the potential role of inflammasomes in neurons, we first examined expression of various inflammasome-related genes. Quantitative-PCR with the Universal ProbeLibrary system was used to detect the mRNA levels of *Nlrp1a*, *Nlrp1b*, *Nlrp1c*, *Nlrp3*, *Nlrp6*, *Nlrp12*, *Nlrc4*, *Aim2* and *Asc*. The results showed that, in cultured mouse cortical and hippocampal neurons, *Aim2* was the most highly expressed inflammasome sensor compared to the other inflammasome sensors (Fig. 1a). This result echoes a previous study showing Aim2 expression in neurons^14^. To further confirm this result, we performed quantitative-PCR using the SYBR Green system with another two different sets of primers for *Aim2. Nlrp3*, the most well-studied inflammasome sensor, was also included to compare with *Aim2*. *Aim2* expression levels were much higher (~10-fold) than those of *Nlrp3* (Fig. 1b). In addition to the cultured neurons, adult mouse brains were also analyzed. Although the expression levels of *Nlrp3* and *Nlrc4* were noticeably increased in adult brains compared with those of cultured neurons, *Aim2* expression levels were still the most elevated among the different inflammasome sensors when detected with the Universal ProbeLibrary system, as well as with the SYBR Green system (Fig. 1c,d). To further confirm the higher expression levels of *Aim2* in the brain, the actual copy number of *Aim2* was measured by absolute quantitative RT-PCR. In adult mouse brains, the copy number of *Aim2* mRNA was estimated to be ~190 per ng of total RNA, which was 6.2- and 18.2-fold higher than those of *Nlrp3* and *Nlrc4*, respectively (Fig. 1e), further supporting the high expression of *Aim2* in the brain. We then monitored the temporal expression of *Aim2* and *Nlrp3* by examining mouse brains at postnatal (P) day 0, 3 and 7, as well as at two months. Compared with P0 and P3, the brains of mice at two months of age had reduced (~50%) expression of *Aim2* (Fig. 1f). In contrast, the mRNA expression level of *Nlrp3* doubled in the brain during development from P0 to two months (Fig. 1g), though its expression level was still lower than that of *Aim2* (Fig. 1e). These expression analyses suggest that AIM2 likely plays a role in brains.

### IL-1β is released by cultured neurons upon TLR7 and AIM2 activation

To examine AIM2 function in neurons, we first monitored IL-1β secretion upon AIM2 activation. Since TLR7 activation increases IL-1β mRNA expression^13^, to maximize the effect of AIM2 activation, we applied CL075 and poly-deoxyadenylic-deoxythymidylic acid (poly dAdT) —the synthetic double-stranded DNA (dsDNA) —to activate TLR7 and AIM2, respectively, in cultured cortical and hippocampal neurons. The amounts of IL-1β in the supernatant of neurons treated with CL075 and poly dAdT increased approximately 5-6-fold when compared with the vehicle control (Fig. 1h). AIM2 activation by poly dAdT alone slightly induced IL-1β expression, but the difference was not significant (Fig. 1h). The combinatory effect of CL075 and poly dAdT on IL-1β production in the cultured neurons required AIM2 because *Aim2*−/− neurons did not produce IL-1β in the presence of CL075 and poly dAdT (Fig. 1h). These results suggest that AIM2 expression enables neurons to produce IL-1β under dsDNA stimulation and TLR7 activation.

### AIM2 regulates neuronal morphology

**Figure 2:**
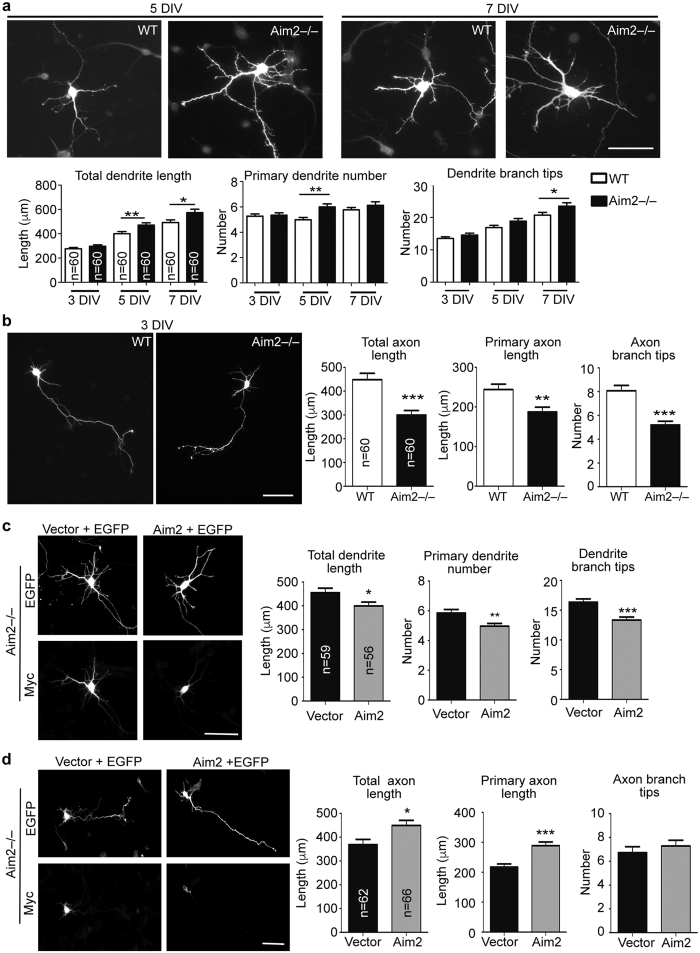
Deletion of *Aim2* inhibits axonal growth but promotes dendritic growth. (**a**,**b**) Cortical and hippocampal mixed cultures prepared from *Aim2*−/− and WT embryos were transfected with EGFP at 1 DIV and subjected to immunostaining using GFP and SMI-312 R (axonal marker) or MAP2 (dendritic marker) antibodies at different time points as indicted. After identifying neurite property (either axons or dendrites) based on SMI-312R or MAP2 signals, the lengths of axons and dendrites were determined by GFP signals. (**a**) Dendritic phenotypes. (**b**) Axonal phenotypes. (**c**,**d**) *Aim2*−/− cortical and hippocampal mixed cultures were transfected with Myc-tagged *Aim2*, EGFP and control vector, as indicated, at (**c**) 2 and (**d**) 1 DIV and harvested for immunostaining at (**c**) 5 and (**d**) 3 DIV. The phenotypes of (**c**) dendrites and (**d**) axons were quantified as indicated. In (**a**,**b**), only GFP images are shown; in (**c**,**d**), both GFP and Myc signals are shown. Scale bar, 50 μm. Neurons were collected from two independent experiments. The sample sizes (n) of examined neurons are indicated. The data represent the mean plus s.e.m. *p < 0.05; **p < 0.01; ***p < 0.001.

A previous study suggested the role of IL-1β in controlling the cell morphology of cultured neurons^38^. It seems possible that AIM2 controls IL-1β secretion and thereby regulates neuronal morphology. We investigated the role of AIM2 in neuronal differentiation and studied whether IL-1β acts downstream of AIM2 in regulating neuronal development. We first manipulated the expression levels of *Aim2* and monitored the dendritic and axonal growth of cultured neurons. Two different experiments were performed. The first experiment was to compare neuronal morphology of wild-type and *Aim2*−/− neurons. Total dendrite length, primary dendrite number and the number of dendritic branch tips were determined at 3, 5 and 7 DIV. The results showed that *Aim2*−/− neurons had longer total dendrite length at 5 and 7 DIV, more primary dendrites at 5 DIV and more branch tips at 7 DIV (Fig. 2a). In measuring axon formation, *Aim2* deletion reduced both primary and total axon length and the number of axonal branch tips at 3 DIV (Fig. 2b). To confirm that the morphological changes in *Aim2*−/− neurons are due to *Aim2* deletion, we reintroduced *Aim2* to *Aim2*−/− neurons. The results showed that restoration of *Aim2* expression reduced total dendrite length, primary dendrite number and the number of dendritic branch tips (Fig. 2c). Similarly, re-expression of *Aim2* had the opposite effect on axonal growth—increasing both total and primary axon length (Fig. 2d). This suggests that the role of AIM2 in neurons is more complex than a simple control of cell growth or death, instead likely involving a specific function to influence dendritic and axonal differentiation.

**Figure 3:**
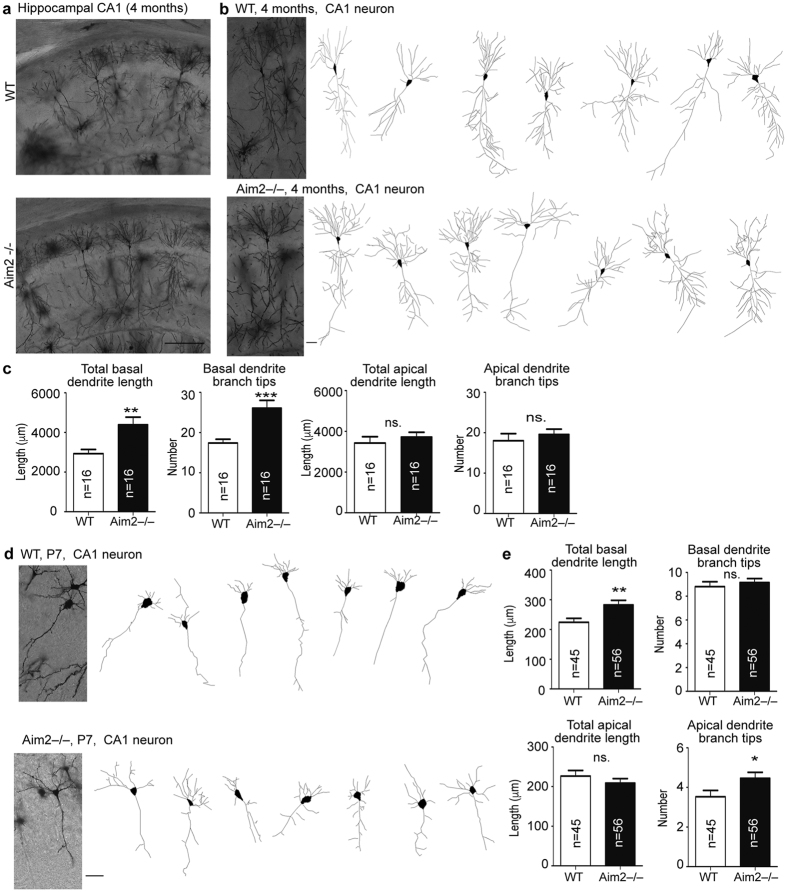
*Aim2* deletion alters dendritic arborization *in vivo*. (**a**) Representative images of hippocampal CA1 region *in vivo* revealed by Golgi staining of adult mice. (**b,d**) Images of individual CA1 neurons. (**c**,**e**) Quantitative results of basal and apical dendrites are shown. (**b,c**) Adult mice; (**d,e**) P7 mice. Neurons were collected from 5–7 neonatal and adult mice for each genotype. The sample sizes (n) of examined neurons are indicated. The data represent the mean plus s.e.m. *p < 0.05; **p < 0.01; ***p < 0.001. Scale bar, (**a**) 200 μm; (**b**,**d**) 50 μm.

The second experiment was to examine the effect of AIM2 *in vivo* using Golgi staining to analyze *Aim2*−/− mouse brains. In four-month-old mouse brains, *Aim2*−/− CA1 neurons exhibited a noticeably different pattern of dendritic arbors compared with WT neurons (Fig. 3a,b). For basal dendrites, both total dendrite length and the number of total dendritic branch tips were noticeably increased in *Aim2*−/− CA1 neurons (Fig. 3c). In contrast, *Aim2* deletion did not influence the length and branching level of apical dendrites (Fig. 3c). Because neonatal brains had higher *Aim2* expression levels compared to adult brains (Fig. 1f), we also analyzed dendritic arbors in P7 brains. Similar to adult brains, total basal dendrite length was increased in *Aim2*−/− CA1 neurons at P7 (Fig. 3d,e), although the total number of branch tips did not differ between WT and *Aim2*−/− brains (Fig. 3d,e). Upon observing apical dendrites, we found that total dendrite length was not noticeably affected by *Aim2* deletion. Nevertheless, the branching level of apical dendrites was higher in *Aim2*−/− CA1 neurons at P7 (Fig. 3d,e). These *in vivo* analyses support the role of AIM2 in controlling neuronal morphology at both neonatal and adult stages.

### Activation of AIM2 inflammasomes influences neuronal morphology

**Figure 4:**
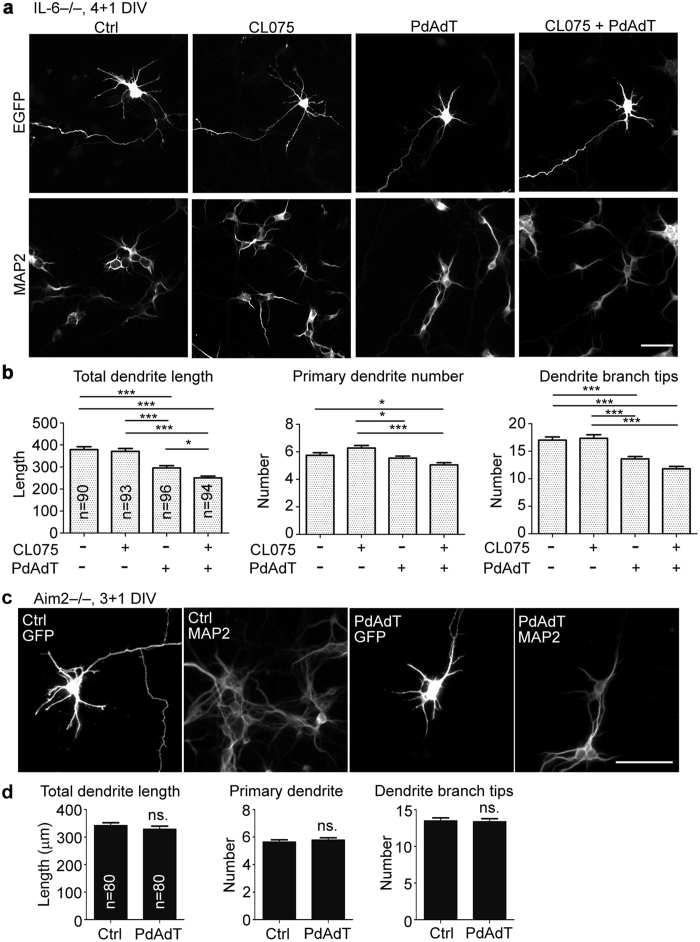
Activation of AIM2 inflammasomes inhibits dendritic growth. (**a**,**b**) Cortical and hippocampal mixed cultures that were prepared from *IL-6*−/− mice were first transfected with EGFP at 2 DIV, treated with CL075 (6 μM) for 6 hours, followed by transfection with poly dAdT (PdAdT, 2 μg) at 4 DIV, and finally fixed at 5 DIV. Fixed neurons were immunostained with EGFP and MAP2 antibody to outline cell morphology and to label dendrites. (**a**) Representative images. (**b**) Quantitative data of total dendrite length, primary dendrite number and the number of dendrite branch tips. (**c**,**d**) *Aim2*−/− neurons do not respond to poly dAdT to regulate neuronal morphology. Cortical and hippocampal mixed cultures prepared from *Aim2*−/− embryos were transfected with EGFP at 2 DIV and transfected with poly dAdT (PdAdT, 2 μg) one day later. Neurons were fixed at 4 DIV for immunostaining using GFP and MAP2 (dendritic marker) antibodies as indicated. (**c**) Representative images (**d**) Dendritic phenotype. Neurons were collected from three independent experiments. The sample sizes (n) of examined neurons are indicated. The data represent the mean plus s.e.m.. Scale bar, 50 μm. *p < 0.05; ***p < 0.001.

In addition to manipulating *Aim2* expression, we also investigated the role of AIM2 in neurons by activating AIM2 inflammasomes. We compared CL075 alone, poly dAdT alone, and a combination of CL075 and poly dAdT to the vehicle control. In our previous study, we showed that TLR7 activation by CL075 induces IL-6 expression, which ultimately restricts dendritic growth^13^. To exclude the contribution of IL-6, *Il-6*−/− cortical and hippocampal neurons were used. Consistent with our previous data^13^, CL075 alone did not alter the dendritic growth of *Il-6*−/− neurons (Fig. 4a,b). When CL075 was combined with poly dAdT, all dendritic parameters, including the total dendrite length, primary dendrite number and the number of dendritic branches, were noticeably reduced in *Il-6*−/− neurons (Fig. 4a,b), which suggests a negative role for AIM2 inflammasomes in dendritic growth. We also noticed that poly dAdT treatment alone was sufficient to downregulate the dendritic growth of *Il-6*−/− neurons (Fig. 4b), although it did not noticeably induce the amount of IL-1β expression observed for cultured neurons (Fig. 1h). To confirm the involvement of AIM2 in the effect of poly dAdT on neuronal morphology, poly dAdT was added into *Aim2*−/− neurons. Addition of poly dAdT did not influence dendritic growth of *Aim2*−/− neurons (Fig. 4c,d). These results suggest that AIM2 senses dsDNA in the environment to regulate morphology of cultured neurons.

### IL-1β signaling is required for AIM2 to regulate neuronal morphology

**Figure 5:**
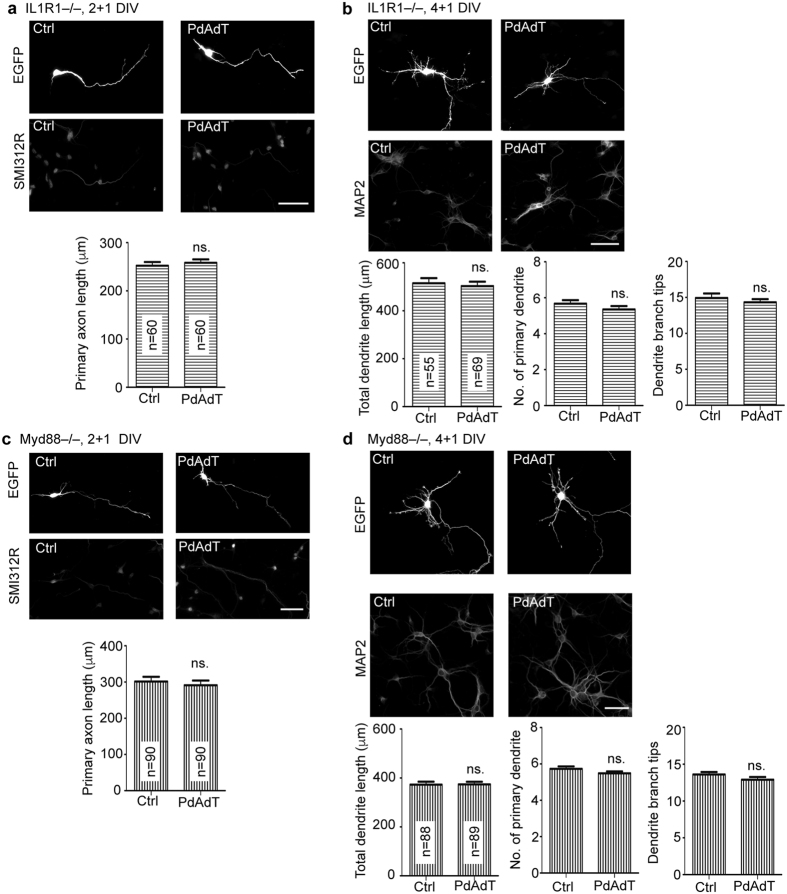
Poly dAdT does not influence the neuronal morphology of *Il1r1*−/− and *Myd88*−/− neurons. Cortical and hippocampal mixed cultures prepared from *Il1r1*−/− and *Myd88*−/− embryos were transfected with EGFP at (**a,c**) 1 and (**b,d**) 2 DIV and transfected with poly dAdT (PdAdT, 2 μg) at (**a,c**) 2 and (**b,d**) 4 DIV. One day later, neurons were fixed for immunostaining using GFP and SMI-312R (axonal marker) or MAP2 (dendritic marker) antibodies as indicated. (**a,c**) Axonal phenotype and (**b,d**) dendritic phenotype were then determined based on GFP signals. Scale bar, 50 μm. The sample sizes (n) of examined neurons are indicated. The data represent the mean plus s.e.m. ns, non-significant.

The aforementioned results suggest that addition of poly dAdT is sufficient to activate AIM2 to restrict dendritic growth but is unable to noticeably increase IL-1β secretion into culture medium (Figs 1h and 4b). It is possible that the amount of IL-1β necessary to restrict dendritic growth was lower than the detection limit of our instruments. Alternatively, in addition to IL-1β, AIM2 might use another effector to downregulate dendritic growth. To investigate whether IL-1β is involved downstream of AIM2 in regulating neuronal morphology, we examined the effect of poly dAdT on two mutant neurons: 1) *Il1r1*−/− neurons, which lack the most well-studied receptor for IL-1; and 2) *Myd88*−/− neurons, which lack the key downstream adaptor molecule MYD88 for IL-1 receptor signaling^39^. Both these mutant neurons are unable to respond to IL-1β. We found that, unlike wild-type neurons, poly dAdT did not influence axonal and dendritic growth of *Il1r1*−/− neurons (Fig. 5a,b). Similarly, *Myd88*−/− neurons also did not respond to poly dAdT in terms of controlling axonal and dendritic growth (Fig. 5c,d). These data support the notion that IL-1β is required for poly dAdT to control neuronal morphology.

### AIM2 activation cell-autonomously regulates neuronal morphology

**Figure 6:**
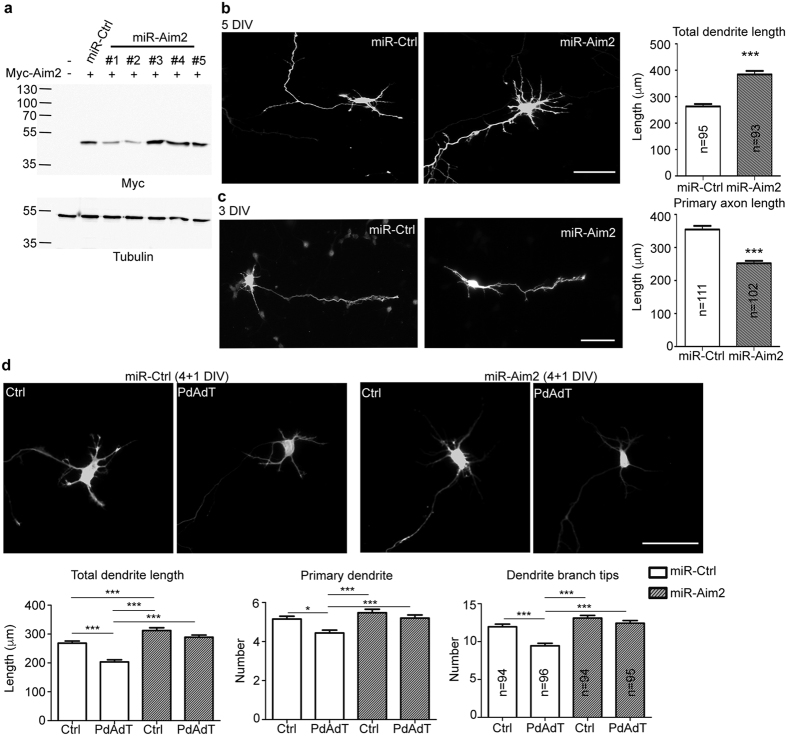
*Aim2* knockdown inhibits axonal growth while promoting dendritic growth in WT cultured neurons. (**a**) Knockdown effect of miR-Aim2 constructs on Myc-tagged *Aim2* expression in HEK293T cells. Five miR-Aim2 constructs were cotransfected with Myc-tagged *Aim2* into HEK293T cells. Immunoblotting was performed using Myc and β-tubulin antibodies. (**b,c**) Cultured cortical and hippocampal neurons were transfected with *Aim2* knockdown construct miR-Aim2 and non-silencing control miR-Ctrl at (**b**) 2 DIV and (**c**) 1 DIV and subjected to immunostaining using GFP and (**b**) MAP2 or (**c**) SMI-312R antibodies. The axonal and dendritic lengths were analyzed based on the EGFP signals. Only GFP images are shown. (**d**) Transfection of miRNA constructs in WT cultured neurons at 2 DIV, followed by transfection with 2 μg poly dAdT (PdAdT) at 4 DIV. Dendrite morphology was analyzed at 5 DIV. Scale bar, 50 μm. The sample sizes (n) of analyzed neurons are indicated. The data collected from three independent experiments are presented as the means plus s.e.m. *p < 0.05; ***p < 0.001.

Although we found that AIM2 responded to poly dAdT stimulation and used the IL-1β pathway to regulate neuronal morphology, our data also show that poly dAdT alone did not significantly increase IL-1β secretion into the culture supernatant. It seems possible that a very low amount of IL-1β is sufficient to regulate neuronal morphology, in which case an autocrine mechanism is likely the most effective way for a low amount of neuronal IL-1β to control neuronal morphology. To investigate whether AIM2 uses a cell-autonomous mechanism to influence neuronal morphology, we reduced *Aim2* expression by transfecting an *Aim2* knockdown construct. In our neuronal culture systems, transfection efficiency is usually less than 1%. Therefore, transfected neurons are surrounded by untransfected neurons, providing a good system to test the cell-autonomous effect of AIM2. If AIM2 acts cell-autonomously to regulate neuronal morphology, reducing *Aim2* expression by an *Aim2* knockdown construct should be sufficient to generate the abnormal neuronal morphology observed for transfected neurons. To investigate this possibility, we first constructed five different artificial miRNA knockdown constructs of *Aim2*. In HEK293T cells, miR-Aim2#2 exhibited the best effect in reducing the protein expression of Myc-tagged *Aim2* (Fig. 6a). MiR-Aim2#2 (termed MiR-Aim2 hereafter) was then transfected into neurons. The results showed that miR-Aim2 expression in neurons resulted in longer total dendritic length and shorter total axonal length (Fig. 6b,c), which is similar to the phenotypes of *Aim2*−/− neurons (Fig. 2). Moreover, if AIM2 activation acts non-cell-autonomously to regulate neuronal morphology, *Aim2* knockdown neurons should still be influenced by IL-1β secreted from neighboring neurons upon poly dAdT treatment. To investigate this possibility, poly dAdT treatment was included. We found that poly dAdT restricted dendritic arborization of neurons transfected with non-silencing control miR-Ctrl, but this effect did not occur in the *Aim2* knockdown neurons (Fig. 6d). Taken together, these results suggest that the effect of poly dAdT on neuronal morphology is mediated by an AIM2-dependent cell-autonomous mechanism.

### Aim2 deletion induces anxious behaviors and impairs auditory fear memory

**Figure 7:**
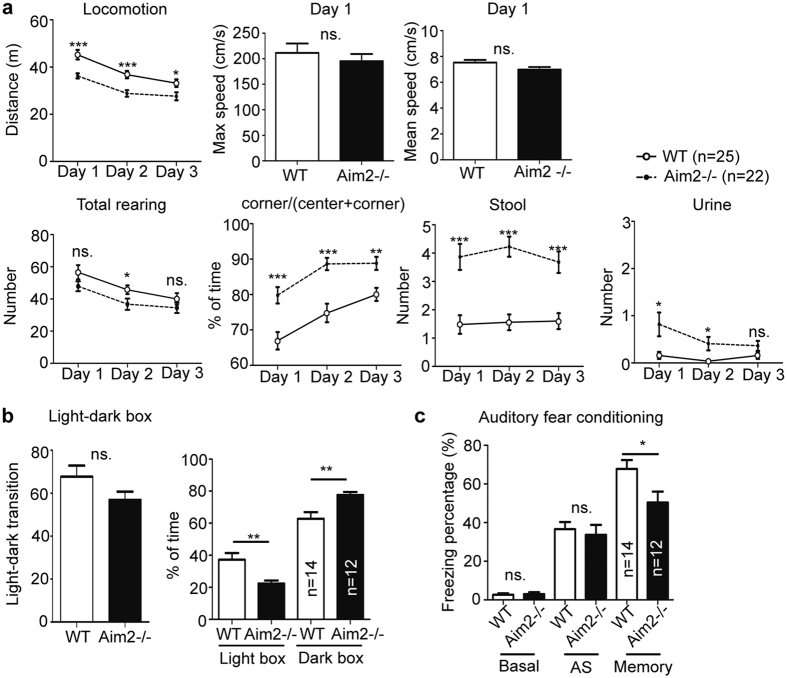
*Aim2* deletion reduces locomotor activity, induces anxious behaviors and impairs associative memory. (**a**) Open field. The total travel distance (locomotion), moving speed, number of total rearing events (exploration), the percentage of time spent at a corner (anxiety), numbers of urine stains and fecal pellets in the arena are shown. (**b**) Light-dark box. The number of transitions between light and dark boxes and the percentage of time spent in the light or dark was analyzed. (**c**) Auditory fear conditioning was analyzed. Basal, the freezing response during habituation. AS, freezing response right after stimulation. Memory, the freezing response one day after training. Sample numbers (n) are indicated in each panel. *Aim2*−/− and WT mice are compared. Data represent mean plus s.e.m. The results of unpaired *t* tests are shown. *p < 0.05; **p < 0.01; ***p < 0.001; ns, not significant.

The above results suggest the role of AIM2 in regulation of neuronal morphology. We then wondered whether AIM2 is relevant for brain functioning. A series of behavioral paradigms, including open field, light-dark box and fear conditioning, were applied to analyze *Aim2*−/− and WT mice. In open field, *Aim2*−/− mice had noticeably lower locomotor activity, as shorter moving distances were recorded on all three examination days (Fig. 7a). Because the moving speed of *Aim2*−/− mice was comparable to that of WT mice (Fig. 7a), the shorter moving distance was not caused by lower moving speed. The total number of rearing events also tended to be lower in *Aim2*−/− mice, but this was only statistically significant for Day 2 (Fig. 7a). We noticed that *Aim2*−/− mice preferred to stay in the corners of their boxes (Fig. 7a), and also defecated and urinated more in the open field (Fig. 7a), both of which are suggestive of anxious behaviors in *Aim2*−/− mice. To confirm this point, we performed light-dark box experiments. Indeed, compared with WT animals, *Aim2*−/− mice spent more time in the dark box, though the numbers of transitions between light and dark boxes were comparable between WT and *Aim2*−/− mice (Fig. 7b). These data support that *Aim2* deletion induces anxiety.

In fear conditioning, the freezing responses during habituation and the period right after foot shocks were comparable between *Aim2*−/− and WT mice (Fig. 7c). However, one day after auditory fear conditioning, *Aim2*−/− mice had a noticeably lower freezing percentage in response to tone stimulus (Fig. 7c), suggesting a role for AIM2 in regulating associative memory.

In conclusion, our analyses suggest that *Aim2* deletion influences brain functions.

## Discussions

In this report, our data suggest that neuronal AIM2 inflammasomes are able to sense dsDNA to promote IL-1β secretion by neurons. IL-1β then acts as an autocrine signaling mechanism to downregulate dendritic growth, whilst promoting axonal growth. In classical innate immune responses, the combinatory signals that activate TLRs and inflammasomes provoke proteolytic processes and massive secretion of the IL-1 family of cytokines^27^,^40^. In cultured neurons, we also found that, by adding exogenous agonists, activation of both TLR7 and the AIM2 inflammasomes promotes IL-1β secretion. However, in the absence of exogenous ligands, manipulation of *Aim2* expression levels in cultured neurons was sufficient to regulate neuronal morphology, which is perhaps due to endogenous ligands released from dead cells or healthy neurons in cultures, echoing previous studies showing that endogenous ligands of TLR7 regulate neuronal death and morphology^13^,^16^,^19^. In this scenario, it is likely that there is a basal level of IL-1β in cultures, which is also able to regulate cell growth of neurons. Manipulation of Aim2 expression levels may influence the processing of this basal level of IL-1β. Since the level of secreted IL-1β is so low, it can only act as an autocrine signal to cell-autonomously control neuronal morphology. This mechanism is likely involved in regulation of neural development *in vivo*. As for our analysis of *Aim2*−/− brains, we found that without any immune challenge, *Aim2*−/− CA1 neurons already had a more complex dendritic arbor at P7. Perhaps, apoptotic cells during development serve as a source of endogenous ligands to activate the AIM2 inflammasomes of neurons. These neuronal innate immune responses allow neurons to detect their growing environment and avoid extending their dendrites to regions with dead cells.

Our study suggests that AIM2 activation restricts dendritic growth but promotes axonal extension. Thus, the effect of AIM2 on neuronal morphology is unlikely to be due to the induction of cell death. In fact, IL-1β has been shown to have a differential effect on dendrites and axons. It can downregulate the dendritic growth of cultured neurons^38^. For axonal growth, however, the reported function of IL-1β is controversial. During the development of sympathetic neurons, 100 ng/ml IL-1β inhibits nerve growth factor-induced axonal growth^41^. In contrast, IL-1β at a concentration of 50 ng/ml stimulates neurite growth of enteric neurons^42^. High doses (500 ng/ml) of IL-1β trigger axonal growth of entorhinal cortex slice cultures, but not transverse spinal cord slice cultures^43^. Thus, it seems likely that different types of neurons respond differently to IL-1β in terms of axonal growth. Nevertheless, taken with our findings, these studies suggest IL-1β may play a role in the regulation of neuronal development, not just induction of pyroptosis (a type of cell death induced by inflammation).

In addition to IL-1β, there are several examples showing that cytokines or growth factors are able to act as autocrine signals regulating neuronal growth. Our previous study showed that TLR7 uses IL-6 to restrict axonal and dendritic growth^16^. Depending on the concentration, IL-6 may act as an autocrine or paracrine signal to regulate neuronal morphology^13^,^16^. Neurons are able to secrete brain-derived neurotrophic factor (BDNF) at the growth cone. Locally-secreted BDNF then cell-autonomously regulates axonal differentiation^44^. Taken together, autocrine signaling may be a general mechanism for neurons to sense environmental cues and to control their growth and differentiation.

Our study shows that *Aim2* deletion induces anxious behaviors and impairs locomotion and associative memory. Previous human genetic and knockout mice have indicated the roles of IL-1 signaling in cognition^45^. The IL-1 receptor is heterodimeric, containing a ligand-binding subunit (IL-1R1) and essential accessory subunits. IL1RAPL1, one of the accessory subunits, regulates postsynaptic signaling^46^ and is associated with mental retardation^46^,^47^. Deletion of *Il1rapl1* in mice results in learning deficiency^48^, which is consistent with our observation that Aim2 deletion impairs auditory fear conditioning. However, in contrast to our finding, reduced anxiety behaviors were found in *Il1rapl1*−/− mice^48^. For *Il1r1*−/− mice, hyper locomotion and decreased anxiety in open field were observed^49^, which also contrast with the phenotype of *Aim2*−/− mice. Several possibilities may explain the conflicting observations in these mutant mice. For instance, there are at least nine different IL-1 receptors and accessory subunits^50^,^51^. Different subunits have different expression patterns and vary in regulation or function. It is not clear if there is complex interplay between these different subunits, which could manifest in altered mouse behavior. It may also explain why many controversial results were observed for the IL-1 study^45^. Moreover, AIM2 is just one of various inflammasome sensors that processes pro-IL-1β and *Aim2* deletion can only impair IL-1β production in response to dsDNA and perhaps only in a subset of cell types. Thus, the impact of *Aim2* deletion and IL-1 receptor deficiency is likely to be different. To resolve this issue, it will be necessary to delete *Aim2* or IL-1 receptors in a cell type-specific manner. Conditional knockout mice are therefore needed to address the contribution of different types of cells to the AIM2-dependent inflammatory response.

## Methods

### Animals

*Il-6*−/− (stock number 002650)^52^, *Aim2*−/− (stock number 013144), *Il1r1*−/− (stock number 003245)^53^ and *Myd88*−/− (stock number 009088)^54^ mice in a C57BL/6 genetic background were purchased from Jackson Laboratory and housed in the animal facility of the Institute of Molecular Biology, Academia Sinica, under a 12 h light/12 h dark cycle. All animal experiments were performed with the approval of the Academia Sinica Institutional Animal Care and Utilization Committee and in strict accordance with its guidelines and those of the Council of Agriculture Guidebook for the Care and Use of Laboratory Animals.

### Chemicals and antibodies

We used rabbit polyclonal GFP (Invitrogen), mouse monoclonal MAP2 (Sigma-Aldrich), mouse monoclonal SMI-312R (Covance), mouse monoclonal Myc (Cell Signaling, 9B11), rabbit monoclonal β-tublin (9F3, Cell Signaling), Alexa Fluor 488- and Alexa Fluor 594-conjugated secondary antibodies (Invitrogen), and HRP-conjugated secondary antibodies (GE Healthcare), as well as CL075 (InvivoGen) and poly dAdT (InvivoGen). Poly dAdT is known to activate AIM2 inflammasomes^14^,^55^,^56^. The length of poly dAdT used in this report is 1184 bps.

### Plasmids

The full-length of the *Aim2* construct, pUNO1-Aim2, was purchased from InvivoGen and further subcloned into GW1-myc vector. *Aim2* miRNA constructs were generated using the BLOCK-iT^TM^ Pol II miR RNA1 Expression Vector Kit (Invitrogen). A plasmid pcDNA 6.2-GW/EmGFP-miR-neg (miR-ctrl, Invitrogen), which was predicted to not target any vertebrate gene, was used as a negative control. The detailed plasmid construction strategy and primer sequences are available in the Supplementary Materials.

### Cell culture, Transfection, Neuronal Morphometry, Q-PCR and ELISA

Cortical and hippocampal neurons were collected from E17.5 mouse embryos and cultured in Neurobasal medium/DMEM (1:1) with B27 supplement, penicillin, streptomycin and glutamine (Invitrogen). For most transfections, the calcium phosphate precipitation method was used as described^57^. For poly dAdT transfection, we used the Lipofectamine 2000 system (Invitrogen). To investigate neuronal morphology, cells were seeded at a density of 2.5 × 10^5^ cells/well in 12-well plates with 18 mm poly-L-lysine-coated coverslips. For ELISA and Q-PCR, cells were seeded at a density of 2.5 × 10^6^ and 1 × 10^6^ cells/well in poly-L-lysine-coated 6-well plates, respectively. The detailed methods for neuronal morphometry, Q-PCR and ELISA^13^ are available in the Supplementary Materials.

### Animals and behavioral analyses

The animals used in the behavioral assays were offspring of Aim2 knockout or wild-type C57BL/6J. Male mice were used for behavioral assays to rule out a hormonal effect of female mice on offspring behaviors. All of the procedures for the behavioral analyses have been described previously^15^,^58^,^59^ with minor modification. Behavioral analyses were performed in sequence as follows: week 12, open field; week 13–14, light-dark box; week 14–16, auditory fear conditioning. The animals were moved from the housing room to the behavioral room for accommodation for 1 week prior to the behavioral tests. Before tests, the mice were put into a new cage for 10 min habituation. After each test, the mice were put into another new cage until all mice had finished the test. Therefore, the tested mice did not affect the non-tested mice. All tests were performed from 14:00 to 19:00. The detailed protocols for each behavioral paradigm are available in the Supplementary Materials.

### Statistical analysis

Statistical analyses were performed using GraphPad Prism. For data in Figs 2,3,5 and 7, an unpaired two-tailed *t*-test was used. For data in Figs 1 and 4, a one-way ANOVA with Bonferroni correction was used. For data in Fig. 6, a two-way ANOVA with variance was used. The data are presented as the mean ± s.d. (Fig. 1) or s.e.m. (remaining figures). For morphological experiments, n indicates the numbers of examined neurons.

## Additional Information

**How to cite this article**: Wu, P.-J. *et al*. AIM 2 inflammasomes regulate neuronal morphology and influence anxiety and memory in mice. *Sci. Rep.* **6**, 32405; doi: 10.1038/srep32405 (2016).

## Supplementary Material

Supplementary Information
