# Supplementary material for: AIM 2 inflammasomes regulate neuronal morphology and influence anxiety and memory in mice
Source: Sci Rep. 2016 Aug 26;6:32405. doi: 10.1038/srep32405 (PMC5000013; doi:10.1038/srep32405)
Supplement: Supplementary Information [file srep32405-s1.pdf]

Supplementary Information for

AIM 2 inflammasomes regulate neuronal morphology and influence anxiety and memory in mice

Pei-Jung Wu[1,2], Hsin-Yu Liu[2], Tzyy-Nan Huang[2], Yi-Ping Hsueh[1,2]*

[1]Graduate Institute of Life Sciences, National Defense Medical Center, Taipei 114, Taiwan.

[2]Institute of Molecular Biology, Academia Sinica, Taipei 115, Taiwan.

*To whom correspondence should be addressed: Dr. Yi-Ping Hsueh, Institute of Molecular Biology, Academia Sinica, 128, Academia Road, Section 2, Taipei 115, Taiwan, Republic of China. E-mail: yph@gate.sinica.edu.tw

Supplementary Methods

Plasmids

The full-length of the *Aim2* construct, pUNO1-Aim2, was purchased from InvivoGen. *Aim2* cDNA was PCR-amplified using a forward primer (5'-GGT ACC CCG AGA GTG AGT ACC GGG-3', where the underlined segment indicates a KpnI site) and a reverse primer (5'-GAAGATCT TCACTCCACACTTTTC-3', where the underlined segment indicates a BglII site), and subcloned into the GW1-myc vector with KpnI and BglII digestion. The plasmid GW1-myc was used as the vector control for GW1-myc-Aim2. For the miRNA constructs, the target sequences of *Aim2* were designed by the BLOCK-iT™ RNAi Designer tool (Invitrogen). The primer sequences are listed as follows:

miR Aim2#1:
top 5'-TGCTGTCAACAACAGCATTTCCCGGTGTTTTGCACTGACTGACACCGGGAAGCTGTTGTTGA-3';
Bottom 5'-CCTGTCAACAACAGCTTCCCGGTGTCAGTCAGTGGCCAAAACACCGGGAAAGCTGTTGTTGAC-3'.
miR Aim2#2:
top 5'-TGCTGTTTCAGTTCTTCCTCCGTGATGTTTTGGCCACTGACTGACATCACGGAACTGAAA-3';
Bottom 5'-CCTGTTTCAGTTCTTTCCGTGATGTCAGTCAGTGGCCAAAACATCACGGAGGAAGAACTGAAAC-3'.
miR Aim2#3:
top 5'-TGCTGTAACTCTGTCCTATCTGCCACGTTTTGGCCACTGACTGACGTGGCAGAGGACAGAGTTA-3';
Bottom 5'-CCTGTAACTCTGTCCTCTGCCACGTCAGTCAGTGGCCAAAACGTGGCAGATAGGACAGAGTTAC-3'.
miR Aim2#4:
top 5'-TGCTGTGAATCAGGTGGTCAGCTAACGTTTTGGCCACTGACTGACGTTAGCTGCACCTGATTCA-3';
Bottom 5'-CCTGTGAATCAGGTGCAGCTAACGTCAGTCAGTGGCCAAAACGTTAGCTGACCACCTGATTCAC-3'.
miR Aim2#5:
top 5'-TGCTGCGCACCTGCACTTTGAATCAGGTTTTGGCCACTGACTGACCTGATTCAGTGCAGGTGCG-3';
Bottom 5'-CCTGCGCACCTGCACTGAATCAGGTCAGTCAGTGGCCAAAACCTGATTCAAAGTGCAGGTGCGC-3'.

The paired oligonucleotides were inserted into a pcDNA 6.2-GW/EmGFP-miR vector using the BLOCK-iT^{TM} Pol II miR RNA1 Expression Vector Kit (Invitrogen). A plasmid pcDNA 6.2-GW/EmGFP-miR-neg (miR-ctrl, Invitrogen), which was predicted to not target any vertebrate gene, was used as a negative control.

Quantitative RT-PCR (Q-PCR)

Q-PCR was performed as described [1]. Briefly, neurons were plated at a density of 1 x 10^6 cells/well in poly-L-lysine-coated 6-well plates and harvested at 4 DIV for RNA extraction. Both cultured neurons and mouse brains were extracted using Trizol reagent according to the manufacturer's instructions (Invitrogen, Carlsbad, CA), followed by DNase I (NEB) digestion for 30 min at 37°C. Three and 5 μg of total RNA purified from cultured neurons and mouse brains, respectively, were used to generate cDNA using the Transcriptor First Strand cDNA Synthesis Kit (Roche) with an oligo (dT)18 primer. Quantitative-PCR was performed using a LightCycler480 (Roche) and Universal ProbeLibrary system (UPL; Roche) or SYBR Green system (Bio-Rad). For UPL, the primers and probes were designed using the Assay Design Center Web Service (http://qpcr.probefinder.com/roche3.html). The PCR thermal program was denaturation at 95℃ for 10 min; 45 cycles of denaturation at 95℃ for 10 sec, annealing at 60℃ for 30 sec, and extension at 72℃ for 1 sec; and a final cooling step at 40℃ for 30 sec. The primer sequences and corresponding probes are as follows:

*Universal ProbeLibrary system:
Nlrp1a-F: 5'-GGAGCCTTGTTCAAAGACACA-3' and Nlrp1a-R: 5'-TTGATCAGAAGTGATAGAGGAGACC-3', with the UPL Probe #68;
Nlrp1b-F: 5'-GAGCTGATGAAGCGCAGAC-3' and Nlrp1b-R: 5'-TGTGTACTTTTGGAATTGTCCCTA-3', with the UPL Probe #106;
Nlrp1c-F: 5'-TGTACCTTTCTCGTTTTGTGTCTC-3' and Nlrp1c-R: 5'-TTGAAAGTGGGCAACATGG-3', with the UPL Probe #17;
Nlrp3-F: 5'-CCCTTGGAGACACAGGACTC-3' and Nlrp3-R: 5'-GAGGCTGCAGTTGTCTAATTCC-3', with the UPL Probe #82;
Nlrp6-F: 5'- CCAGCTTCTGCATCTGAGAGT-3' and Nlrp6-R: 5'-CTCCCTTGCCACTGCATC-3', with the UPL Probe #15;
Nlrp12-F: 5'-CACCAGACCTGCAGACTCC-3' and Nlrp12-R: 5'-CATGCTTTGGAGGTGAGTCC-3', with the UPL Probe #5;
Aim2-F: 5'-TGGGCTGTTTAAAGTCCAGAA-3' and Aim2-R: 5'-CACCTCCATTGTCCCTGTTT-3', with the UPL Probe #6;
Nlrc4-F: 5'-TGTGATCTCCAAGAGATGAAGTTG-3' and Nlrc4-R: 5'-GATCAAATTGTGAAGATTCTGTGC-3', with the UPL Probe #40;
ASC-F: 5'CCCTTGTTCGTCTACCCTCA-3' and ASC-R: 5'-GGAACAGTTAAGCGCCAAAG-3', with the UPL Probe #110;
Cyp-F: 5'-TGCCCAGCAGTTTAGTACCC-3' and Cyp-R: 5'-TGCTTCCCTGTCTCCACAGT-3', with the UPL Probe #64;
Gapdh-F: 5'-AATGTGTCCGTCGTGGATCT-3' and Gapdh-R: 5'-CCCAGCTCTCCCCATACATA-3', with the UPL Probe #80;
*SYBR Green system:
Aim2_1-F: 5'-AAGAGAGCCAGGGAAACTCC-3', Aim2_1-R: 5'-TGTCTCCTTCCTCGCACTTT-3';

Aim2_2-F: 5'-TCTCCTTCCTCGCACTTTGT-3', Aim2_2-R: 5'-TGAAGATTCAGCCATGTGGA-3';
Nlrp3_1-F: 5'-ATGCTGCTTCGACATCTCCT-3', Nlrp3_1-R: 5'-AACCAATGCGAGATCCTGAC-3';
Nlrp3_2-F: 5'-TACGGCCGTCTACGTCTTCT-3', Nlrp3_2-R: 5'-CGCAGATCACACTCCTCAAA-3'.

To measure actual copy number of Aim2, Nlrp3 and Nlrc4, absolute quantitative RT-PCR was performed as described [1,2]. Briefly, the plasmids containing *Nlrp3* and *Nlrc4* coding sequences were first constructed into TA cloning vector pDrive (Qiagen) using RT-PCR cloning with the following primer pairs: (1) Nlrp3-F: 5'-CCCTTGGAGACACAGGACTC-3'; Nlrp3-R: 5'-ACCTCACAGAGGGTCACCAC-3'; based on the DNA sequence with accession No. NM 145827. (2) Nlrc4-F: 5'-GGGAGGGGATGGACTACATA-3'; Nlrc4-R: 5'-TCATTCCCATCCTTTTCCAG-3'; based on the DNA sequence with accession No. NM 001033367. A 10-fold serial dilution of the pDrive-*Nlrp3*, pDrive-*Nlrc4* and pUNO1-m*Aim2* (InvivoGen), ranging from 1.52 nM to 0.00152 nM, was used to create the standard curves for *Nlrp3*, *Nlrc4* and *Aim2*. The actual copy numbers of *Aim2*, *Nlrp3* and *Nlrc4* transcripts in total mouse brain RNAs were then determined by the calculation described in a previous study [2].

Enzyme-Linked Immunosorbent Assay (ELISA)

At 4 DIV, neurons were treated with CL075 for 6 h, followed by poly dAdT treatment for 4 more hours. Culture supernatants were collected and analyzed using the mouse IL-1β ELISA Set (eBioscience) based on the manufacturer's instructions. Each treatment was undertaken in triplicate in a single experiment and the experiments were repeated three times.

Immunofluorescence staining

Primary neurons were fixed with 4% paraformaldehyde and 4% sucrose in phosphate buffered saline (PBS) for 15 min at room temperature. After a wash with PBS three times, cells were permeabilized with 0.2% Triton-X 100 in PBS for 10 min at room temperature and blocked with 5% bovine serum albumin (BSA) in PBS for 60 min. Neurons were then incubated with primary antibodies that were diluted in 5% BSA in PBS (1:200) overnight at 4℃. Neurons were subsequently washed with 0.2% Tween 20 in PBS three times, and then incubated with Alexa Fluor 488- and Alexa Fluor 594-conjugated secondary antibodies (1:500) for 1 h at room temperature. Counter-staining was performed with DAPI. Neuronal images were visualized and recorded at room temperature using a fluorescence microscope (Axioimage M2; Zeiss) equipped with a 20x/NA 0.80 (Plan-Apochromat) objective lens and a cooled charge-coupled device camera (Rolera EM-C^2; QImaging) driven by Zen software (Zeiss). For publication, images were processed using Photoshop with minimal adjustments to contrast or brightness applied to entire images.

Neuronal morphology

To determine neuronal morphology, an EGFP construct was transfected alone or with

other plasmids into cultured neurons at 1 DIV for axon analysis and at 2 DIV for dendrite analysis. Two (for axon) or three (for dendrite) days later, neurons were harvested for immunostaining. Axons were identified with the axonal marker SMI-312R and dendrites were identified with the dendritic marker MAP2. Once processes had been identified (i.e. as either axon or dendrite), the morphological features of processes of individual neurons were then determined by the signals of EGFP. Three parameters were used to monitor axonal morphology as follows. (1) The primary axonal length. The longest axon was recognized as the primary axon; the remainder was considered axonal branches. The length starting from the base of an axon (the site attached to the soma) to the tip was determined based on EGFP signal. (2) The total axonal length. Both the primary axon and axonal branches were measured based on EGFP signal. (3) The axonal branch tips, based on the total number of axonal termini. For dendrites, three parameters were determined: (1) the number of primary dendrites, where the primary dendrites are the processes directly emerging from the soma with a length > 10 μm; (2) the total dendrite length, including primary dendrites and all dendritic branches; and (3) the dendritic branch tips, based on the total number of dendritic termini. Image quantitation was performed using ImageJ software version 1.47v without any additional plugins. Based on the microscope settings, each pixel represents 0.32 μm for the digital images acquired by a 20X lens. This parameter was set in ImageJ using "Set scale" under "Analyze". "Segmented line" combined with "Measure" was then used to determine the actual lengths of axons and dendrites. The data were then exported to Excel and GraphPad Prism for further quantitative and statistical analyses. To minimize bias, other lab members relabeled the samples to perform a blinded test.

Golgi staining

Brains that were collected from P7 and adult (4 months) male mice were immersed in Golgi-Cox solution (SolutionA+B mixture, FD Rapid GolgiStain kit; FD 148 NeuroTechnologies, Columbia, MD, USA) and kept away from light at room temperature for four days. The brains were then transferred into solution C and stored away from light in a cool room for two more days. They were then sectioned into 150 μm-thick coronal sections with a Vibratome sectioner (Leica). The sections were mounted on gelatin-coated slices, rinsed with distilled water and developed in the mixture of solution D + E. The sections were visualized using an AxioImager M2 microscope (Carl Zeiss) that was equipped with a 159 20×/NA 0.8 (M27) objective lens (Plan Apochromat; Carl Zeiss) and captured with an AxioCam MRm digital camera that was operated using Zen Blue software. Camera lucida drawings were performed using ImageJ software (1.47v) and Photoshop (Adobe).

Immunoblotting

HEK293T cells were lysed in RIPA buffer [150 mM NaCl, 50 mM Tris-HCl (pH 7.4), 1% TritonX 100, 0.25% sodium deoxycholate, 0.1% SDS, 2 mM EDTA and 1 mM PMSF] at 4℃ for 30 min. After centrifugation in a microcentrifuge at 13,000 rpm for 30 min at 4℃, the protein concentrations of supernatants were determined using a Bio-Rad protein assay kit (Bio-Rad Laboratories). Proteins were separated by SDS-

PAGE and then transferred to PVDF membranes (Millipore) at 200 mA for 2 h. Finally, the membranes were blocked with 5% non-fat milk in PBS for 1 h at room temperature and incubated with primary antibody in blocking buffer overnight at 4°C. The membranes were then washed three times with 0.1% Tween 20 in PBS and incubated with HRP-conjugated secondary antibodies for 1 h at room temperature. Finally, the signals were developed with ECL plus reagent (Advansta) and captured using an ImageQuant LAS4000 biomolecular imager (GE).

Open field

The apparatus for the open field test was an open transparent plastic box (40 x 40 x 32.5 cm). Four equal-size squares were marked in the corners of the box. The total area of the four corners was equal to the area of the central square region. For the open test, mice were placed individually in the central square region of the box, and their movements were recorded by video camera from the top once a day in 10 minute sessions for 3 consecutive days at the same time each day. After the tests, the mice were returned to their respective home cages and the numbers of urine stains and fecal pellets in the box were counted. All video was analyzed using the Smart Video Tracking System (Panlab, Barcelona, Spain).

Light-dark box

The apparatus for this test was modified from the open field box. An open black box (19 x 39 x 45 cm) was inverted and put into the open field box to divide it into two equal size compartments. A small opening (5 cm in diameter) at the bottom of the black box allowed movement of mice between the two compartments. An individual mouse was placed into the light compartment and then allowed to explore the apparatus for 10 min. The movement was recorded by video camera and the percentage of time in the light box was analyzed using the Smart Video Tracking System (Panlab, Barcelona, Spain).

Fear conditioning

Before training, mice were pre-trained in a habituation chamber (Box B, 15 x 15 x 19.5 cm,) for two days (D-1, -2) to habituate them to the operator and equipment. On the training day (D0), mice were first placed in the training box (Box A), which consisted of a transparent plastic box with sidewalls and a ceiling (15 x 15 x 19.5 cm) for 4-mins habituation (basal responses). After 4-mins habituation, mice received three tone-shock pairings, comprising three repeats of a tone (conditioned stimulus, CS; 18 s, 2kHz) followed by an electronic foot shock (unconditioned stimulus, US; 0.6 mA, 2 s) with a 1-minute inter-trial interval. After training, mice were returned to their home cage. At day 1 (D1), mice were placed in Box B and received 20 tones (20 s,2 kHz) with 5-second inter-trial intervals to observe the freezing response to auditory stimulation. The freezing percentages responding to the first five tones were averaged to reflect auditory fear conditioning. Freezing behaviors were measured

using the FreezeScan^{TM} 2.0v system (CleverSys Inc.). The floor of the chamber was composed of stainless steel grids, which were used to deliver a foot shock of 0.6 mA. The chamber was placed in a sound-attenuating box, which was illuminated by a 7.5 W white light. A constant background noise of 60 dB was produced by ventilation fans inside the box. The chamber was cleaned with 70% ethanol between each mouse. Before tests, Box A was swabbed with 70% ethanol and then 0.1 % acetic acid. Box B was swabbed with 70% ethanol only.

Reference

1 Liu, H. Y. *et al.* TLR7 negatively regulates dendrite outgrowth through the Myd88-c-Fos-IL-6 pathway. *J Neurosci* 33, 11479-11493 (2013).

2 Whelan, J. A., Russell, N. B. & Whelan, M. A. A method for the absolute quantification of cDNA using real-time PCR. *J Immunol Methods* 278, 261-269 (2003).
